# Unveiling the Silent Danger of Childhood Obesity: Non-Invasive Biomarkers Such as Carotid Intima-Media Thickness, Arterial Stiffness Surrogate Markers, and Blood Pressure Are Useful in Detecting Early Vascular Alterations in Obese Children

**DOI:** 10.3390/biomedicines11071841

**Published:** 2023-06-26

**Authors:** Monica Simina Mihuta, Corina Paul, Andreea Borlea, Cristina Mihaela Roi, Oana-Alexandra Velea-Barta, Ioana Mozos, Dana Stoian

**Affiliations:** 1 Department of Doctoral Studies, Victor Babes University of Medicine and Pharmacy, 300041 Timisoara, Romania; simina.mihuta@umft.ro (M.S.M.); cristina.cepeha@umft.ro (C.M.R.); 2 Center of Molecular Research in Nephrology and Vascular Disease, Faculty of Medicine, Victor Babes University of Medicine and Pharmacy, 300041 Timisoara, Romania; borlea.andreea@umft.ro (A.B.); stoian.dana@umft.ro (D.S.); 3 Department of Pediatrics, Victor Babes University of Medicine and Pharmacy, 300041 Timisoara, Romania; 4 2nd Department of Internal Medicine, Victor Babes University of Medicine and Pharmacy, 300041 Timisoara, Romania; 5 3rd Department of Odontotherapy and Endodontics, Faculty of Dental Medicine, Victor Babes University of Medicine and Pharmacy, 300041 Timisoara, Romania; velea.oana@umft.ro; 6 Department of Functional Sciences—Pathophysiology, Center for Translational Research and Systems Medicine, Victor Babes University of Medicine and Pharmacy, 300173 Timisoara, Romania; ioanamozos@umft.ro

**Keywords:** cardiovascular risk, carotid intima-media thickness, central blood pressure, childhood obesity, increased blood pressure, pulse wave velocity, subclinical atherosclerosis

## Abstract

Obese children present a higher cardio-metabolic risk. Measuring vascular biomarkers that assess the evolution of arterial stiffness, subclinical atherosclerosis, and hypertension in such patients could be helpful in the long term. We studied 84 children, aged from 6 to 18 years: 50 obese subjects, versus 34 of normal weight. Clinical examination involved: BMI, waist circumference, waist-to-height ratio, and detection of the presence of acanthosis nigricans and irregular menstrual cycles (the latter in adolescent girls). The carotid intima-media thickness (CIMT) was measured with the Aixplorer MACH 30 echography device. The pulse wave velocity (PWV), augmentation index (AIx), and peripheral and central blood pressures (i.e., SBP, DBP, cSBP, cDBP, and cPP) were acquired through a Mobil-O-Graph device. Obese subjects underwent body composition analysis with a Tanita BC-418. Blood tests were: HOMA-IR, lipid panel, uric acid, and 25-OH vitamin D. All vascular biomarkers presented increased values in obese subjects versus controls. The following cut-off values were significant in detecting obesity: for PWV > 4.6 m/s, cSBP > 106 mmHg for the <12-year-olds, PWV > 4.5 m/s and cSBP > 115 mmHg for the 12–15-year-olds, and PWV > 5 m/s, cSBP > 123 mmHg for the >15-year-olds. AIx is higher in obese children, regardless of their insulin resistance status. Waist circumference and waist-to-height ratio correlate to all vascular parameters. HOMA-IR is an independent predictor for all vascular parameters except CIMT. Cut-off values for PWV of >4.8 m/s, SBP > 125 mmHg, and a cSBP > 117 mmHg predicted the presence of acanthosis nigricans. Obese girls with irregular menses displayed significantly higher PWV, SBP, and DPB. Elevated levels of uric acid, LDL-c, non-LDL-c, triglycerides, and transaminases, and low levels of HDL-c and 25-OH vitamin D correlated with higher arterial stiffness and CIMT values. We conclude that CIMT and the markers of arterial stiffness are useful in the early detection of vascular damage in obese children.

## 1. Introduction

Childhood obesity is a significant preventable health problem requiring continuous attention and effort. The latest statistics show that, in the European Region, around 60% of adults and 29% of school-aged children are overweight or obese [1,2]. In the United States, the prevalence of obesity in children older than two is 19.7% [3]. During the *SARS-COV-2* pandemic, body mass index rates nearly doubled [4], with notable increases in overweight and obesity rates across different age groups [5]. 

Obesity requires a comprehensive framework encompassing prevention and treatment to avoid long-term complications, particularly when it begins, in its early stages. Overlaying complications include metabolic syndrome, metabolic associated fatty liver disease, polycystic ovary syndrome (PCOS), and sleep apnea, all of which contribute to vascular damage [6,7,8]. 

Our aim is to address the early stages of atherosclerosis, a dangerous complication of excess weight. Detecting visible signs of vascular disruptions is crucial for effective management of childhood obesity and its long-term vascular consequences. Our previous research has linked intima-media thickness [9], pulse wave velocity, and blood pressure to childhood obesity [10]. This study focuses on providing additional data on subclinical atherosclerosis and arterial stiffness in the same type of individuals. 

Atherosclerosis is caused by lipid deposits in arterial walls and is driven by pro-inflammatory signaling and immune cell infiltration. Low-density lipoproteins (LDL-c) contribute to endothelial lipid accumulation. Atherosclerosis is most prominent near arterial bends and bifurcations with altered blood flow [11]. Excess adipose tissue in obesity triggers chronic inflammation, disrupting the balance of nitric oxide and constricting hormones [12]. This impairs vasodilation, damages arterial elasticity, and is worsened by high blood pressure, insulin resistance/glucose intolerance, and dyslipidemia [13,14]. Arterial stiffness, as influenced by age and various factors, is significantly aggravated by childhood obesity, which is probably the most important preventable cause [13].

Carotid intima-media thickness (CIMT) measures the gap between the intimal and medial layers of an artery, indicating generalized atherosclerosis [14,15]. It is a non-invasive marker observed through ultrasonography, and is commonly used for cardiovascular diagnosis and risk assessment in adults [14,15]. In obese children, CIMT values are higher compared to normal-weight peers and serve as predictors of future cardiovascular diseases [16,17]. 

Arterial stiffness can be assessed using two markers: pulse wave velocity (PWV) and augmentation index (AIx). PWV measures the speed of the pulse through the arterial tree, reflecting the elasticity of large arteries and predicting cardiovascular events [18,19,20]. Obese children have higher PWV, which is strongly correlated with BMI and waist circumference [21,22,23]. AIx evaluates small artery elasticity by estimating the augmentation caused by the reflected wave during systole [24,25]. In adults, the AIx predicts organ damage in cardiovascular conditions [26]. However, the AIx in children is influenced by height and sympathetic nervous system activity [27,28]. Small heights are associated with a higher AIx [27]. The intensified activity of the sympathetic nervous system is a coping mechanism in obese children, and seems to be associated with a lower AIx [27,28]. In our previous work regarding arterial stiffness in obese children, we could not determine a consistency in the causal relation between weight excess or cardio-metabolic risk factors and a higher value of AIx, and concluded that more research was needed [29].

Higher blood pressure in obese children (i.e., peripheral BP > 95th percentile) worsens atherosclerosis and arterial stiffness [30,31]. Elevated peripheral BP is associated with higher CIMT and PWV [32,33]. In adults, central BP (cSBP and cDBP) correlates more strongly with vascular markers such as CIMT and left ventricular mass [34,35]. The severity of obesity directly relates to increased central BP in children and youth [36,37,38]. Central pulse pressure (cPP), calculated as the difference between cSBP and cDBP, is also higher in obese children [29,39,40]. Although cPP may predict hypertension and arterial stiffness, caution is advised when using peripheral or central PP as surrogate markers, due to a significant level of mismatch between PP and arterial stiffness [41]. However, when both cPP and PWV are elevated, cardiovascular risk significantly increases [41].

The value of generally evaluating the CIMT and arterial stiffness in children at risk does not assume a diagnostic of atherosclerosis, the detection of atherosclerotic plaques, or a finding of extremely elevated levels for arterial stiffness surrogate markers. As clinicians, we do not expect to find pathological values in our determinations when evaluating obese children with no other chronic diseases. We propose this practice in order to more accurately evaluate the cardiovascular status of these patients right from the start and to provide a more efficient long-term medical management plan in order to prevent the progression of atherosclerosis and avoid cardiovascular events in young adults. Moreover, measuring these vascular biomarkers at young ages increases awareness among pediatric patients and their families about cardiovascular risk, opening new channels of doctor-patient communication relevant to the patient’s goals for a healthier future.

## 2. Materials and Methods

The study involved 84 children aged from six to eighteen and was conducted from June 2022 to December 2022 at the Pediatric Endocrinology Department of the “Pius Brinzeu” Emergency County Hospital in Timisoara.

The patients who were addressed to our department were asked to participate in the study if they presented chronic primary obesity (BMI > 95th percentile), or if they were overweight according to their BMI (>85th percentile), but had a waist circumference (WC) >90th percentile, and did not present other comorbidities. The patients who presented with known contributors to secondary obesity (e.g., endocrinological disorders such as hypothyroidism, type 2 diabetes mellitus, hypothalamic injury/disorders, Cushing syndrome, genetic disorders such as Prader–Willi Syndrome, ghrelin–leptin dysfunction, and use of medication that can induce weight gain such as glucocorticosteroids, sulphonylureas, tricyclic antidepressants, and anti-psychotics) [42,43], or conditions that might lead to increased arterial stiffness (e.g, acute illnesses with systemic inflammatory components, chronic kidney disease, congenital heart disease, oral conditions, familial hypercholesterolemia, vasculitis, and type 1 and 2 diabetes mellitus) were excluded [44]. Fifty patients met the criteria and were included in the present study. Thirty-four age-matched, normal-weight (BMI ranging between percentiles 5 and 85) volunteers made up the control group. The exclusion criteria were applied to them as well. All children included were Caucasian, 86% were of Romanian ethnicity, 12% were of Hungarian ethnicity, and 2% were of Serbian ethnicity.

The University of Medicine and Pharmacy Victor Babes Timisoara’s Ethics Council for Scientific Research approved the study, in accordance with the Helsinki Declaration. Prior to being included in the study, the patients and their legal guardians were informed of all the medical procedures that would take place. Informed consent was signed by all legal guardians of minors and by all 18-year-old patients.

### 2.1. Physical Examination

The physical examination of our patients included measurements of their weight, height, and waist circumference. After calculating the body mass index (BMI, kg/m^2^), the 2022 BMI-for-age extended growth charts were used in order to establish percentiles for BMI [45,46]. The cut-off values proposed by Zong et al. were used in order to establish the percentiles for WC [47]. The waist-to-height ratio (WHR) was also calculated, mindful that a WHR > 0.6 is considered at risk for metabolic syndrome [48]. The puberty development of each subject was established according to Tanner stages [49]. Menstruating girls were asked whether they had regular or irregular menses. Irregular menses were considered menstrual cycles of <21 or >35 days, or missing ≥3 menses in a row, in girls who had their menarche at least 2 years before [50]. The presence of acanthosis nigricans was noted in the patients who displayed it [51]. 

These formulas were used for the calculation of:

❖ BMI = Weight (kg)/Height^2^ (m) [45]
❖ WHR = WC (m)/Height (m) [48]

### 2.2. Body Composition Analysis

The subjects included in the obese group underwent a body composition analysis through body impedance with the Tanita Body Composition Analyzer BC-418 device MA III (T5896, Tokyo, Japan). The device works through 8 electrodes which provide a high-frequency current of 50 kHz, 500 μA that moves through the fingertips of both hands and the tips of the toes of both feet. The voltage is measured on the thenar region of both hands and on the heel region of both feet, respectively. This means that the device has two handles and a support for each foot.

**Figure A1:**
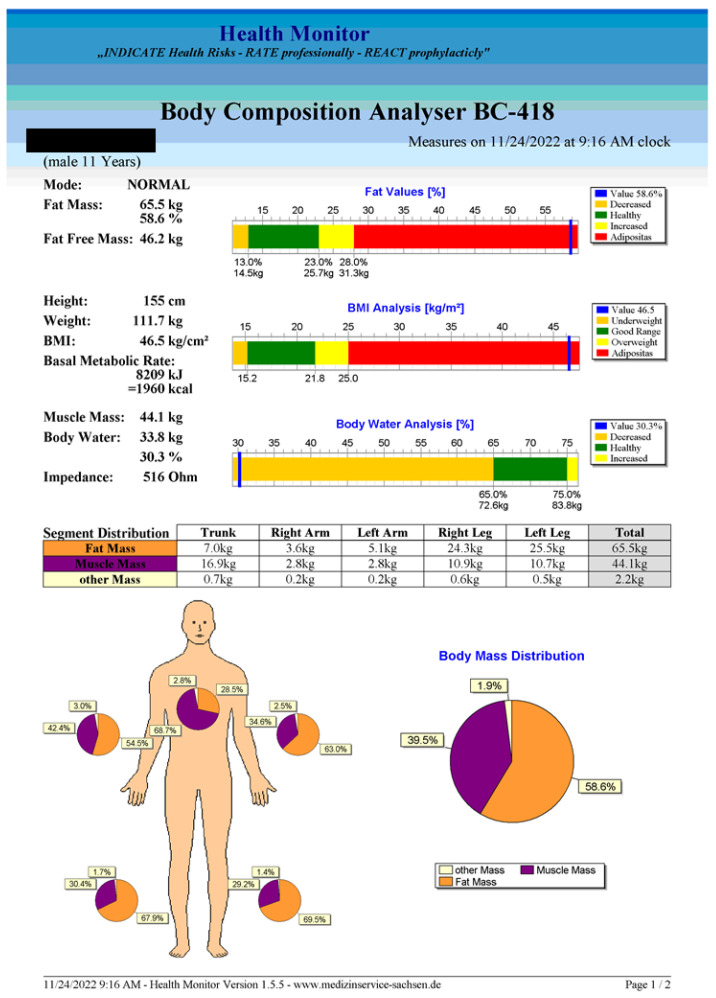
Example of a Body Composition Analysis report for an 11-year-old boy.

The following procedures are necessary in order to obtain correct results:

❖ According to the manufacturer, the use of this device is advised only for children over 6 years old;
❖ The software requires the examiner to enter the following data for each patient: identification data, birth date, sex, weight, height, and clothes’ weight;
❖ The subject steps with bare feet on the device’s foot electrodes, making sure that their feet are positioned exactly in the pre-determined shapes, and then grabs both handles, which contain the hand electrodes;
❖ The subject needs to maintain a stable and straight position throughout the measurement;
❖ After the measurement is accomplished, the software provides an explicit report (an example is given in Figure A1, Appendix A).

The device uses the body’s bioelectrical impedance to accurately estimate body composition, including fat mass and muscle mass. The concept is based on the electricity’s characteristics of passing easily through water, and with difficulty through fat tissue. Hence, the electrical resistance is a measure of how difficult it is for electricity to travel through a substance, and measurements of this resistance can be used to determine the amounts of fat and other body components. The parameters used in this study were the fat mass (kg), trunk fat mass (kg), muscle mass (kg), and body water (kg).

### 2.3. Arterial Stiffness Measurements

The Mobil-O-Graph^®^ 24 Hour ABPM oscillometric arteriograph (M26101200, IEM^®^ GmbH, Stolberg, Germany) was used to acquire the parameters known as biomarkers of arterial stiffness: PWV (m/s), AIx (%), heart rate (HR, beat/minute), and peripheral and central BP (mmHg), as well as the mean arterial pressure (MAP) and central PP (mmHg). The instrument’s one-time measurement feature was utilized.

Details of the methodology and specifics of the device have been presented in our previous work as well [10,40].

We performed a single-point brachial measurement on the naked left upper arm of each subject under the following conditions:

❖ There had been no ongoing acute illness, and no exposure to smoke for at least four hours prior to the measurements, with no consumption of caffeinated drinks allowed for at least a day prior, and a full eight-hour night sleep was had the night before the measurement;
❖ Ten-minutes rest in a supine position was taken prior to the measurement;
❖ The appropriate cuff size had to be chosen for each patient according to the measurement of their upper arm: extra small: 14–20 cm, small: 20–24 cm, medium: 24–32 cm, and large: 32–38 cm;
❖ The measurement was made in the supine position and the subjects needed to be still, silent, and relaxed;
❖ If the measurement’s accuracy was declared subpar by the device’s software, we retook it after a five-minute pause [10,40].

The Mobil-O-Graph may be used in children older than three, according to the manufacturer, after multiple validating studies [52,53,54]. Our team has previously used the device on both children and adults [10,20,40,55]. It is a device well accepted by children, easy to use, and one with reproducible results. 

### 2.4. The Carotid Intima-Media Thickness Measurement

The carotid ultrasonography was carried out using the Aixplorer MACH 30 echography device (Country-SuperSonic Imagine, Aix-en-Provence, France). The SL 10-2 (2–10 MHz) or the SL 18-5 (5–18 MHz) linear ultrasound probes were used, depending on the volume of adipous tissue in the cervical region of each subject. The echography machine software (SuperSonic Imagine 3.0, Aix-en-Provence, France) is able to calculate the CIMT values automatically.

❖ The ultrasonography technique

**Figure A2:**
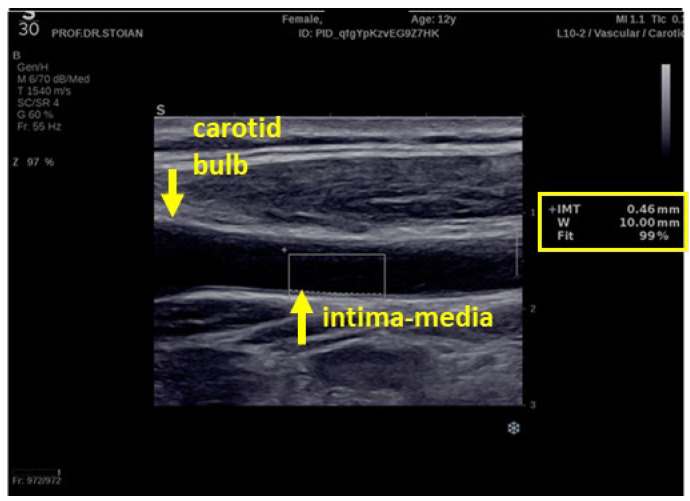
Example of a CIMT measurement for a 12-year-old obese girl (from authors’ collection).

An experienced and certified sonographer performed the carotid ultrasonography on each patient. The subject was asked to lie down and extend their neck backward as much as possible in order to facilitate a better view of the common right and left carotid arteries. The examiner selected the appropriate ultrasound settings (carotid evaluation in B-mode) and the appropriate ultrasound probe. Starting from the clavicle upwards, on each side, the scanning was transversal in order to locate the carotid bulb and the sequential bifurcation of the common carotid into the internal and external carotids. Once the bulb was located, the probe orientation was changed to longitudinal scanning: the carotid was seen in a longitudinal section, with the carotid bulb on the left side of the screen and the image clear enough to distinguish the intima-media. The region recommended for the measurement of the CIMT is located on the posterior carotid wall of the carotid segment, 1–2 cm caudally from the carotid bulb, as exemplified in Figure A2 (see Appendix A) [56,57]. The images were acquired synchronously with an ECG assay, and the CIMT measurement was made during the end-diastole [58]. The software performed the automatic measurement of the CIMT [59]. For each patient, we performed 3 measurements on each carotid artery and used the mean values in our study.

### 2.5. Blood Tests

The following blood tests were obtained: serum insulin, fasting glucose, Homeostatic Model Assessment for Insulin Resistance (HOMA-IR), uric acid, creatinine, lipid profile (total cholesterol, LDL-c, HDL-c), triglycerides, thyroid-stimulating hormone, free T4, 8 a.m. serum cortisol, aspartate aminotransferase, alanine aminotransferase, ionized calcium, and 25-OH vitamin D. The blood samples were taken within a maximum of 2 weeks after the clinical consultation in the Pediatric Endocrinology Department and processed within the hospital’s accredited laboratory. The patients were advised to abstain from consuming foods for at least 12 h prior to their blood tests. The blood tests were scheduled within the 7:30-8:30 a.m. interval.

The following formula was used for calculating the HOMA-IR:

❖ HOMA-IR = [Glucose (mg/dl) × Insulin (µU/mL)]/405 [60].

Due to the fact that HOMA-IR is influenced by puberty development, for the analysis of the obese group concerning the insulin-resistance status, the cut-off values for HOMA-IR were considered to be the following:

❖ For pre-pubertal children (Tanner stage 1) the HOMA-IR cut-off was considered 2.3, and for pubertal children (Tanner stages 2, 3, and 4), 3.4, as defined in a large study on Italian Caucasian obese children aged 8–15 [61];
❖ For post-pubertal children (Tanner stage 5), the HOMA-IR cut-off remains 3.4, as a study on normal-weight and obese children aged 2–17.8 years old placed the cut-off value for obese children at 3.42, at the 75% percentile, for individuals that may develop cardiovascular risk factors very early in life [62].

### 2.6. Statistical Analysis

Microsoft Excel was used for data collection and MedCalc Statistical Software version 20.111 (MedCalc Soft-ware Ltd., Ostend, Belgium) and DATAtab: Online Statistics Calculator (Graz, Austria) were used for the statistics.

The analysis focused on the relationships between clinical and paraclinical parameters (CIMT, arterial stiffness parameters, and blood tests). The normality of data distribution was tested by the Shapiro–Wilk test. Consequentially, we performed statistical tests appropriate for the normality of the data in question: means, Student’s *t*-test, and Pearson’s correlations for the normally distributed variables and medians, the Mann–Whitney test, and Spearman’s correlation for the non-normal ones. The significance threshold was considered *p*-value < 0.05. One-way ANOVA post hoc tests were performed when three groups were compared, including Bonferroni-adjusted *p*-values. ROC-AUC analysis was performed to evaluate the sensibility and specificity of the vascular biomarkers in relation to the presence of obesity and acanthosis nigricans. Multilinear regressions were performed in order to detect which of the clinical parameters and which of the blood tests could act as independent predictors of the vascular biomarkers. 

## 3. Results

Two groups under study were created, one including the obese patients and the overweight who displayed a WC > the 90th percentile (*n* = 50), and one including the normal-weight volunteers (*n* = 34). Table 1 depicts the distribution of the subjects included in the two groups by sex (male or female) and age (<12 years old, 12 to 15 years old, or >15 years old). No differences in mean age were detected between the two groups; the mean age in the obese group was 12.5 (SD = 3.28), while in the control group, it was 12 (SD = 3.61), *p* = 0.48.

Comparisons between all boys and all girls included in the study showed that CIMT values were significantly higher in boys, with a median value of 0.45 mm, compared to 0.43 mm in girls (*p* = 0.01). Other significant differences were not detected in the rest of the vascular biomarkers. We also did not detect any differences between obese girls and obese boys in any vascular parameter.

Regarding differences in the mean values of the vascular parameters between the three age groups which included obese patients, we performed one-way ANOVA post hoc tests, Bonferroni-adjusted, and we detected the following significant results: CIMT was higher in subjects aged from 12 to 15 years than in those <12 years old (*p* = 0.02); PWV and cSBP were higher in >15-year-olds than in <12-year-olds (*p* = 0.01 and *p* = 0.02, respectively). No differences were detected across groups for AIx (*p* = 0.31), HR (*p* = 0.33), SBP (*p* = 0.59), DBP (*p* = 0.35), MAP (*p* = 0.09), cDBP (*p* = 0.2), and cPP (*p* = 0.59). See Table 2.

The obese subjects were divided into subgroups according to the Tanner stages: pre-pubertal (Tanner 1), pubertal (Tanner 2, 3, and 4), and post-pubertal (Tanner 5). The ANOVA analysis detected significant differences across the three groups in all vascular biomarkers except for HR (see Table 3). No significant differences were detected between HR values after the Bonferroni correction.

### 3.1. The Vascular Biomarkers in Relation to the Weight Excess

#### 3.1.1. The Vascular Biomarkers and the BMI, WC, and WHR

The significant differences noted between the two groups of study with regard to the subject’s weight excess, as clinically objectified through the BMI, WC, and WHR, are depicted in Table 4.

**Figure 1:**
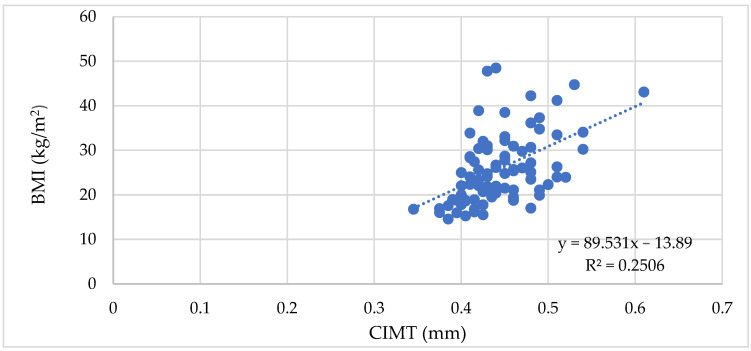
Correlation between the BMI and CIMT in all patients (ρ = 0.52, *p* < 0.0001).

**Figure 2:**
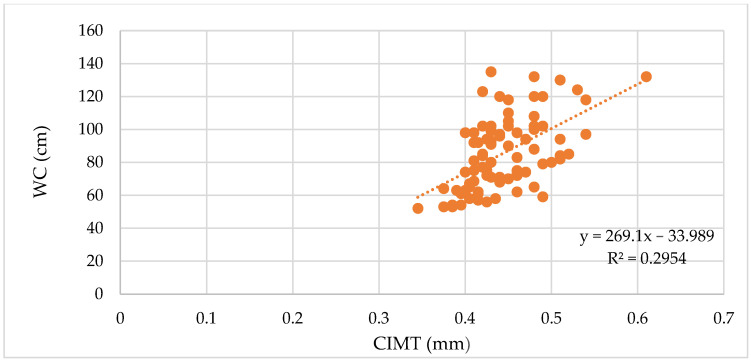
Correlation between the WC and CIMT in all patients (ρ = 0.53, *p* < 0.0001).

**Figure 3:**
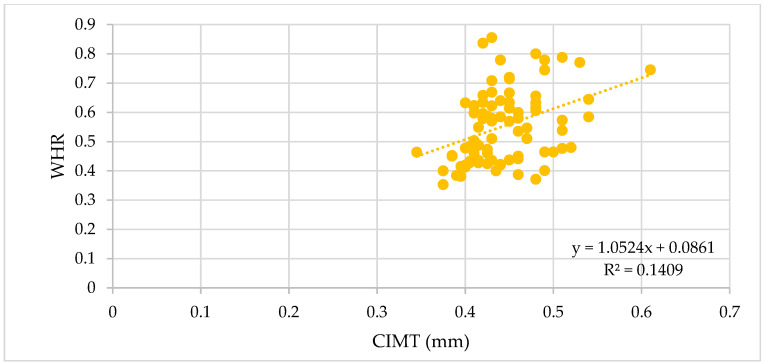
Correlation between the WHR and CIMT in all patients (ρ = 0.38, *p* = 0.0004).

The correlation analysis between BMI, WC, and WHR and the vascular biomarkers revealed significant positive correlations with the vascular biomarkers measured, as depicted in Table 5, with the strongest correlations observed for PWV, SBP, and cSBP. For CIMT 1, AIx, MAP, cDBP, and cPP, we observed moderate correlations (Figure 1, Figure 2 and Figure 3).

The clinical parameters—Tanner stages, clinical adiposity indicators (W, BMI, WC, and WHR), and the presence of acanthosis nigricans—were included as independent factors in a multiple regression to determine which of them would be significant predictors of the vascular biomarkers. In the model regression, the vascular parameters each served as a dependent variable. The most significant variances explained by clinical parameters for the value of a vascular parameter were observed in the case of PWV (64.09%), SBP (62.81%), and cSBP (67.21%), whereas the least significant variance was noted for HR (6.92%). Table 6 presents the model for each regression. 

Furthermore, the multiple regressions detected significant independent predictors of peripheral and central BP values, as depicted in Table 7. Although the clinical markers of adiposity showed significant correlations to CIMT, PWV, and AIx (Table 5), the regressions did not confirm them as independent predictors for these vascular markers. The presence of acanthosis nigricans was not confirmed as a significant predictor, either. The level of pubertal development (Tanner stages) does not present a relationship with CIMT, PWV, and AIx independently of the markers of adiposity, but represents a significant predictor for central BP values. 

#### 3.1.2. The Vascular Biomarkers and the Body Composition Analysis

The body composition analysis performed on the obese subjects showed reliable correlations between the vascular parameters measured and the fat, muscle, and water masses of each patient, as depicted in Table 8. The BMI and fat mass of the obese patients were strongly positively correlated (ρ = 0.87, *p* < 0.0001), as were the WC and trunk fat mass (ρ = 0.78, *p* < 0.0001). The WHR and trunk fat mass were moderately correlated (ρ = 0.37, *p* = 0.008).

#### 3.1.3. Comparisons of Vascular Markers between Obese and Normal-Weight Subjects

**Figure 4:**
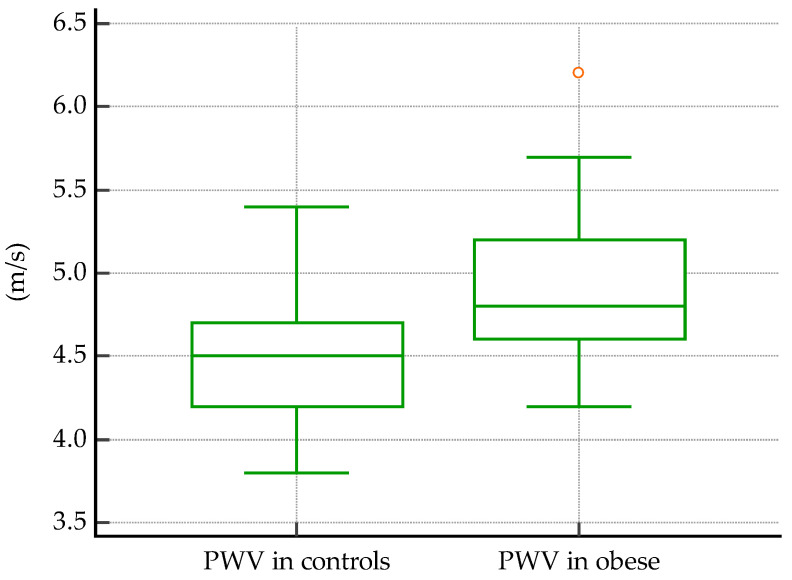
PWV median values in controls vs. obese subjects (*p* < 0.0001).

**Figure 5:**
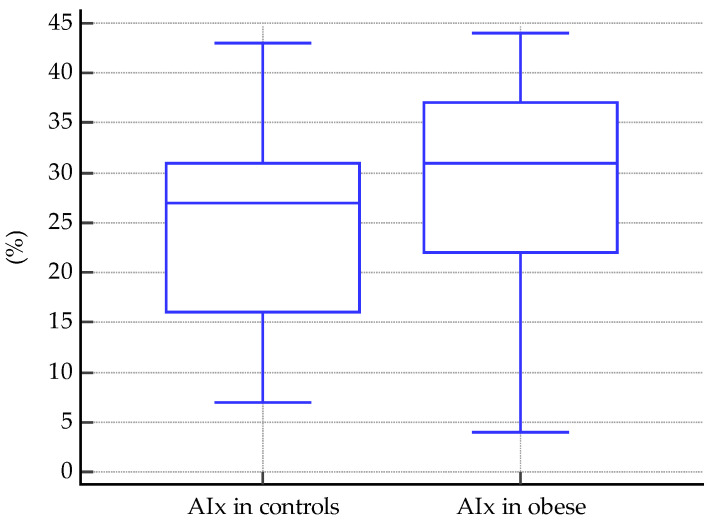
AIx mean values in controls vs. obese subjects (*p* = 0.03).

Significant differences were detected when comparing the vascular biomarkers of obese subjects vs. controls. For PWV, median values were 4.8 m/s in obese subjects, and 4.5 m/s in controls, *p* < 0.0001 (Figure 4). The same was found for AIx, with a mean value of 29.28% in obese subjects and 24.58% in controls, *p* = 0.03 (Figure 5).

**Figure 6:**
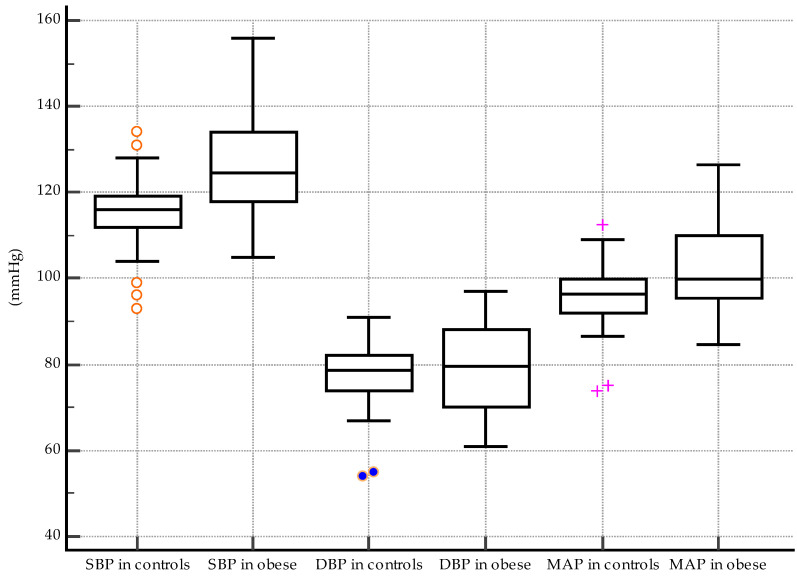
Differences between peripheral BP values: significantly higher SBP (*p* < 0.0001) and MAP (*p* = 0.008) in obese subjects vs. controls (the different symbols accompanying the box-and-whisker plots in the figure suggest that different variables are being compared in the three pairs).

Median values for SBD were significantly higher in obese subjects, 124.5 mmHg vs. 116 mmHg in controls, *p* < 0.0001 (Figure 6). However, the differences between DBP median values were not significant between the two groups (79.5 mmHg in obese subjects and 78.5 mmHg in controls, *p* = 0.63, Figure 6). The analysis showed significant differences between the median values of MAP in obese and normal-weight subjects (100 mmHg and 96.5 mmHg, respectively, *p* = 0.008). (As depicted in Figure 6.)

**Figure 7:**
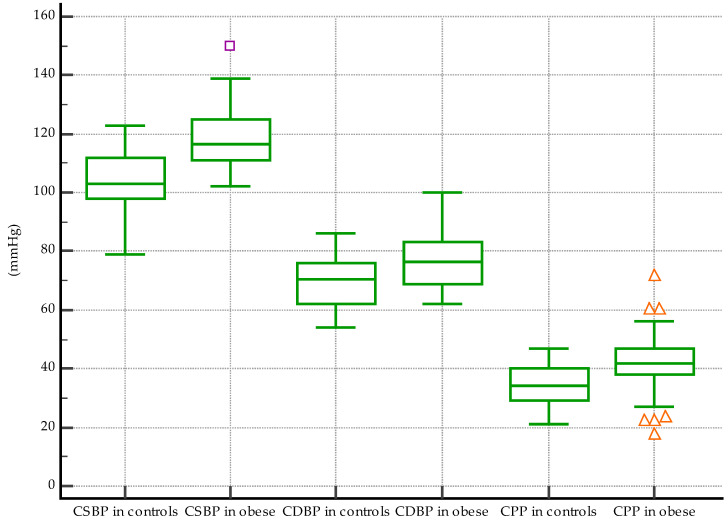
Differences between central blood and pulse pressure values: significantly higher cSBP (*p* < 0.0001), cDBP (*p* = 0.003), and cPP (*p* = 0.001) in obese subjects vs. controls (the different symbols accompanying the box-and-whisker plots in the figure suggest that different variables are being compared in the three pairs).

Furthermore, the analysis showed significantly higher cSBP median values in obese children, 116.5 mmHg, than in controls, 103 mmHg (*p* < 0.0001), see Figure 7. The same was observed in the case of cDBP: a mean value of 77.08 mmHg in obese subjects vs. 69.52 mmHg in controls, *p* = 0.003 (Figure 7). Median values for cPP were also significantly higher in the obese group: 41.7 mmHg vs. 33.4 mmHg, *p* = 0.0001 (Figure 7). 

With regard to HR, significantly higher values were detected in obese children: 91 beats/minute vs. 82 beats/minute (*p* = 0.02).

#### 3.1.4. Predictive Cut-Off Values for the Vascular Biomarkers

❖ Predictive cut-off values for the vascular biomarkers in all subjects

**Figure A3:**
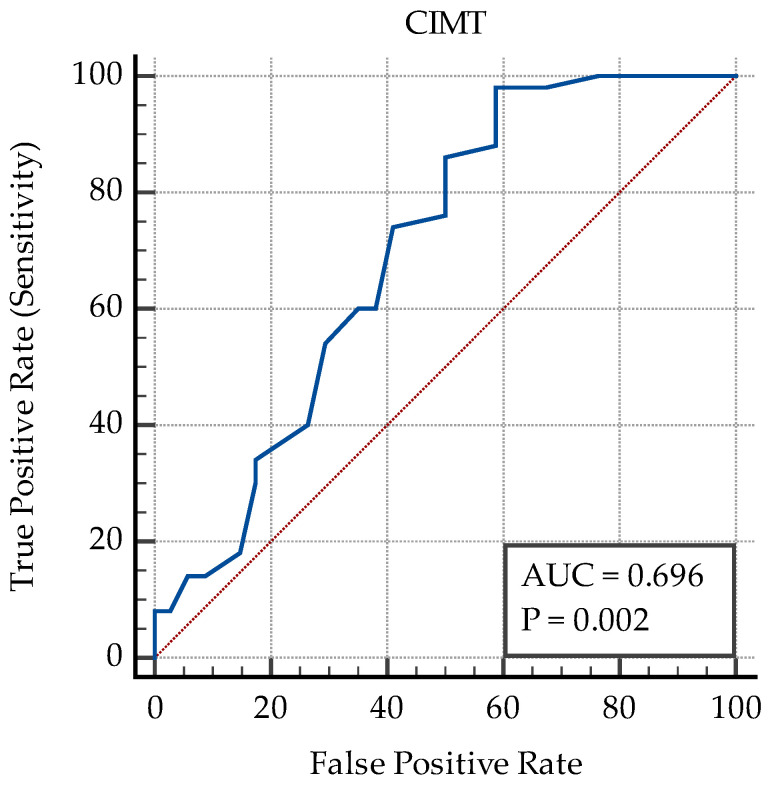
ROC curve for CIMT measurements (CIMT ROC curve is represented in blue).

**Figure A4:**
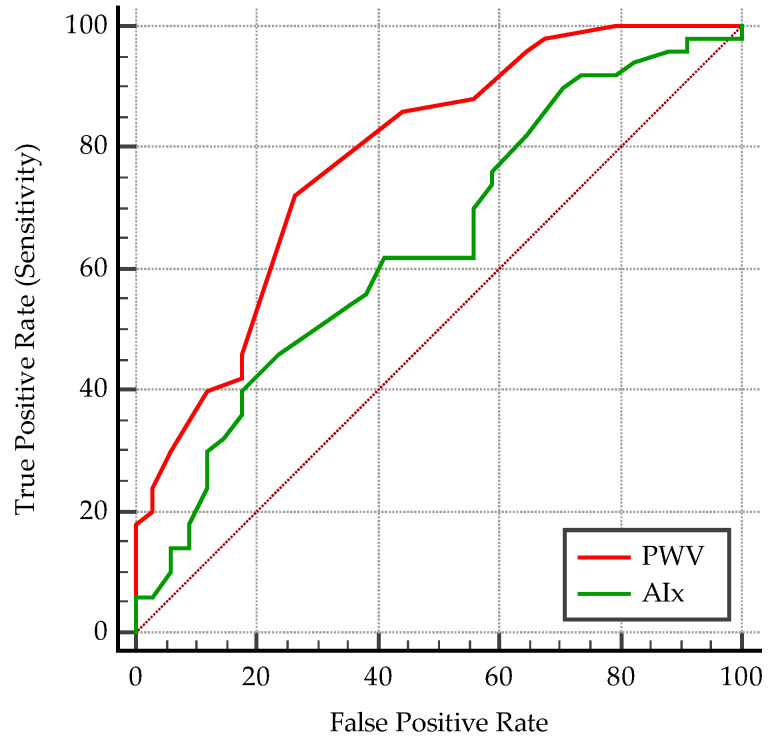
ROC curves for PWV and AIx.

**Figure A5:**
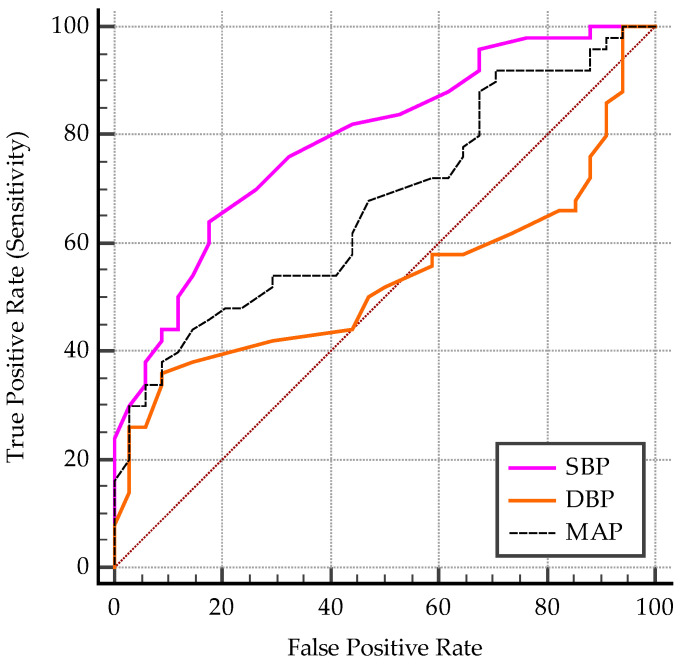
ROC curves for SBP, DBP, and MAP (the red dashed line is the line of equality, otherwise known as the random chance line).

**Figure A6:**
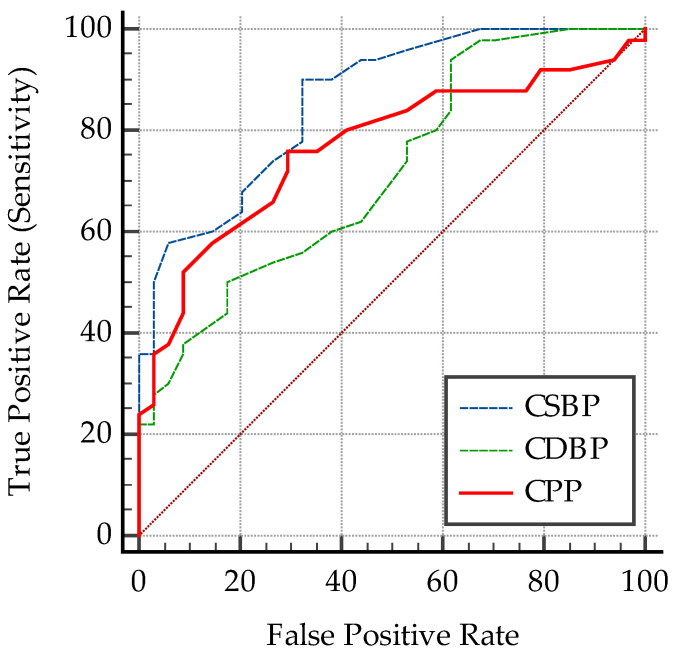
ROC curves for cSBP, cDBP, and cPP (the red dashed line is the line of equality, otherwise known as the random chance line).

The AUC-ROC analysis was made for each vascular parameter, in order to detect the presence of obesity. The optimal cut-off values, defined as the highest sum of sensitivity (Se) and specificity (Sp) for predicting the presence of obesity in children, are depicted in Table 9, and the ROC curves are graphically represented in Figure A3, Figure A4, Figure A5 and Figure A6 (see Appendix A).

❖ Predictive cut-off values for the vascular biomarkers according to age groups

The AUC-ROC analysis was performed on each of the age groups in order to evaluate cut-off values that discriminate between obese and normal-weight children (Table 10).

### 3.2. The Vascular Biomarkers and the Glucose Metabolism

#### 3.2.1. HOMA-IR–A Biomarker of Insulin Resistance

**Figure 8:**
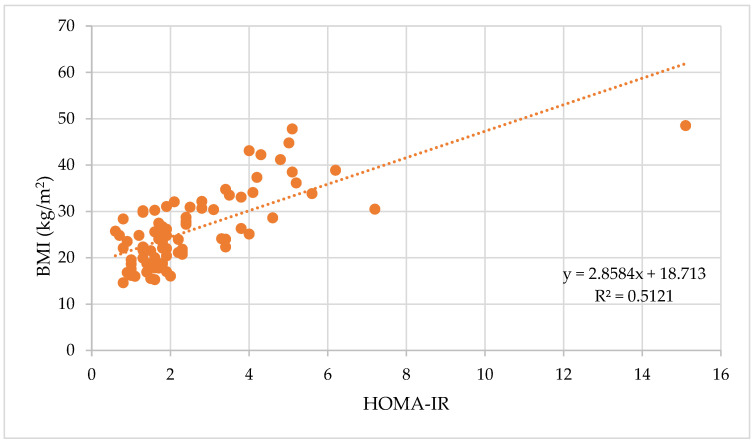
Correlation between HOMA-IR values and BMI.

The HOMA-IR index is strongly positively correlated with the BMI, ρ = 0.66, *p* < 0.0001 (*n* = 84), as depicted in Figure 8. HOMA-IR is also strongly correlated with WC (ρ = 0.69, *p* < 0.0001) and WHR (ρ = 0.51, *p* < 0.0001).

**Figure A7:**
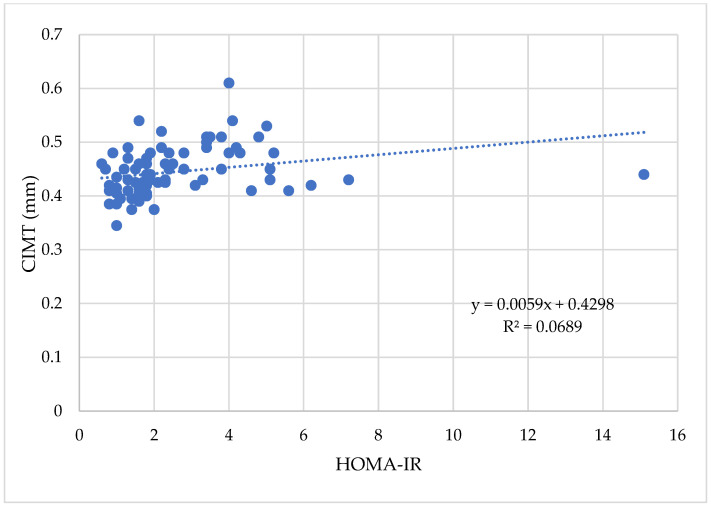
Correlation between HOMA-IR values and CIMT (ρ = 44, *p* < 0.0001).

**Figure A8:**
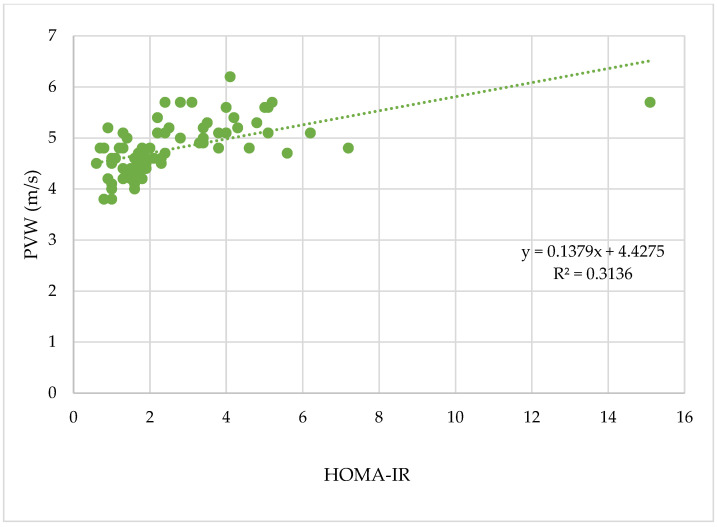
Correlation between HOMA-IR values and PWV (ρ = 64, *p* < 0.0001).

Extremely significant positive correlations were found between the HOMA-IR and the values of vascular markers, as depicted in Table 11 and Figure A7 and Figure A8 (see Appendix A).

Significantly higher values for HOMA-IR were observed in obese pre-pubertal and post-pubertal subjects, compared to their normal-weight peers with the same puberty development (Table 12).

Significant correlations between HOMA-IR values and the vascular biomarkers, according to the aforementioned Tanner stages, were detected in the pre-pubertal and post-pubertal subjects (Table 13). 

#### 3.2.2. Acanthosis Nigricans—A Clinical Sign of Insulin Resistance

Out of the 50 obese subjects, 24 (48%) presented the clinical sign of acanthosis nigricans, while none of the normal-weight subjects presented it. 

The cut-off value for HOMA-IR for obese pre-pubertal subjects was set to 2.3. Out of the twelve subjects (Tanner 1), five (41.6%) presented HOMA-IR values ≥ 2.3. Out of these subjects, three presented acanthosis nigricans. For the Tanner 1 obese patients who did not present HOMA-IR values higher than the cut-off value (seven subjects), only one presented acanthosis nigricans (Table 14). 

The cut-off value for HOMA-IR for obese pubertal and post-pubertal subjects was set to 3.4. Out of the 38 subjects (Tanner 2–5), 39.5% presented a HOMA-IR ≥ 3.4. Out of these subjects, 86.6% presented acanthosis nigricans. Meanwhile, of the obese subjects presenting a HOMA-IR < 3.4, only 30.4% presented acanthosis nigricans. These particular subjects displayed a mean HOMA-IR value of 2.1 (SD = 0.8); see Table 14.

The AUC-ROC analysis made for the vascular parameters, with the presence/absence of acanthosis nigricans being the dichotomous variable, showed an optimal cut-off value for PWV at >4.8 m/s (AUC = 0.77, Se = 75%, and Sp = 80.8%). Also, an SBP value >125 mmHg (AUC = 0.79, Se = 75%, Sp = 84.6%) and a cSBP > 117 mmHg (AUC = 0.8, Se = 70.8%, Sp = 80.8%) are predictive of the presence of acanthosis nigricans. See Table 15.

### 3.3. The Vascular Biomarkers in Relation to the Menstrual Cycle Regularity

The subgroup of girls associated with Tanner stage 5 was evaluated with regard to their menstrual cycles. According to the regularity of their menses, the obese girls were divided into two subgroups: girls with regular menstrual cycles and girls with irregular ones. No girl included in the normal-weight group reported irregular menses (Table 16). 

Although the subgroups are very small (only six subjects in each one), we performed comparison T-Student tests for the vascular biomarkers between the obese girls with regular and irregular menses. The girls who reported irregular menstrual cycles presented significantly higher values for PWV, SBP, DBP, MAP, and cSBP (see Table 17).

### 3.4. The Vascular Biomarkers in Relation to the Blood Tests

The blood test analysis showed significantly higher values in the obese group for the following markers: LDL-c, TG, GPT, GOT, creatinine, uric acid, and ionized calcium (see Table 18).

The correlation analysis detected multiple significant, moderate, and strong correlations between the evaluated blood tests in the obese group, as depicted in Table 19.

A multilinear regression which included all blood parameters and HOMA-IR as independent variables was performed in order to detect which of them would be significant predictors of the vascular biomarkers. The vascular parameters acted in turn as dependent variables in the model regression. The significant independent predictors of vascular biomarkers are represented in Table 20.

## 4. Discussion

This research emphasizes early vascular evaluation in children with obesity, a common cardiovascular risk factor. Despite public health efforts, obesity prevalence in children continues to rise, leading to increased cardiovascular issues in young adults. Therefore, pediatric specialists should consider non-invasive vascular biomarkers for improved diagnosis and management of patients at risk. These biomarkers help detect arterial wall changes before any significant narrowing and impairment occur.

This study focuses on two of the three most important non-invasive markers of atherosclerosis: pulse wave analysis and intima-media thickness. The marker that could not be included in this particular study, flow-mediated dilatation, or nitric oxide-mediated dilation, assesses the earliest alterations in endothelial dilatation function [63]. Pulse wave analysis assesses the intermediate stages of atherosclerosis, evaluating both the function and structure of the blood vessels, by estimating the stiffness of the arteries. The CIMT, which evaluates the structural alterations of the arterial wall, measures subclinical atherosclerosis. Measurements of peripheric and central blood pressure levels represent additional valuable vascular biomarkers, which are intricately involved in the progression of atherosclerosis. 

Undoubtedly, our opinion that obese children present vascular disruptions earlier than their normal-weight peers accords with that of most researchers. The more severe the excess in weight, the higher the values of all the vascular biomarkers measured; strong, significant positive correlations were detected between each vascular parameter and BMI, WC, and WHR (see Table 5). Although BMI is a widely used tool, in both children and adults, for diagnosis of overweight patients and patients with obesity, it does have a serious limitation in its ability to detect body fat percentage. Discrepancies regarding the detection of obesity in different genders, ages, and races are also worrying. However, using BMI in varying combinations with WC and WHR reliably increases the accuracy of health evaluations [64]. This is the reason why we used all three clinical markers of adiposity in our study, in addition to performing bioimpedance body composition analysis for the obese subjects.

With regard to PWV and BP levels, although many studies [22,23,24,65,66,67], including our own [10,40], have shown increased values in children with excess adiposity, the contradictory results of Jakab et al. must be mentioned, as their study included 6816 children [68]. Nevertheless, our results showed significantly higher mean values for CIMT, PWV, AIx, peripheral and central BP, and central PP in obese children. Bittencourt et al. recently published their results, also obtained through a Mobil-O-Graph device, on 5 to 12-year-old children, proposing PWV > 4.09 m/s and cSBPc >  86.17 mmHg as cut-off values for discriminating between obese and non-obese patients [39]. In contrast, our proposed cut-off values for the entire cohort are PWV > 4.6 m/s, Aix > 31%, SBP > 119 mmHg, MAP > 102.5 mmHg, cSBP > 106 mmHg, cDBP > 76 mmHg, and cPP > 36 mmHg. After the AUROC analysis according to age groups, we propose PWV > 4.6 m/s and cSBP > 106 mmHg for the group age <12 years old, a PWV > 4.5 m/s and cSBP > 115 mmHg for the group age 12 to 15 years old, and PWV > 5 m/s and cSBP > 123 mmHg for the subjects older than 15 years old. Sensibility and specificity values, as well as other significant vascular biomarkers according to age groups, can be observed in Table 9 and Table 10. 

In a study similar to ours, Lentferink et al. used a SphygmoCor tonometry device on 62 obese children and adolescents aged from 9 to 19 years old, compared to normal-weight controls, and showed that their average PWV = 4.1 m/s was significantly higher than the PWV of normal-weight subjects [67]. In our study, the mean PWV of the obese group was 4.8 m/s, significantly higher than in controls (*p* < 0.0001, Figure 4). Furthermore, Lentferik et al. also showed that AIx is significantly higher in obese children with insulin resistance than without [67]. In contrast, we showed that AIx is higher in obese children, with or without insulin resistance (*p* = 0.03, Figure 5).

A very interesting prospective study on obese adolescents conducted over a 5-year period showed that, although the BMI of the subjects increased similarly in both obese and controls, over the course of 5 years, the PWV increased by 25% in the obese subjects, while only by 3% in the lean ones. Similarly, the DBP levels grew by 23% in the obese, compared to 6% in the controls [69]. 

Concerning CIMT, cut-off values for the pediatric population are still not standardized, but most studies show that obesity and metabolic syndrome are promoters of an increased CIMT, mainly due to inflammation processes. Farello et al. showed that obese children display higher CIMT regardless of their metabolic status. This team also showed that a higher CIMT in a child with metabolic syndrome represents a significant predictor for cardiovascular events in early adulthood [17]. A very important study on 3497 children aged 6–17 years from five cross-sectional studies conducted on subjects from Brazil, China, Greece, Italy, and Spain showed that CIMT is higher in metabolically healthy obese and overweight children [70]. Our findings confirm the significantly higher values for CIMT among obese children. The results propose a CIMT > 0.4 mm for discriminating obesity in children under 12 years old and >0.46 mm for adolescents (see Table 10). 

The fact that obese children and adolescents display higher peripheral BP values than their lean peers is not a novelty. Obesity in children promotes hypertension through adipokines such as leptin, increasing sympathetic nervous system (SNS) activity [71]. SNS activation constricts renal vascular beds and stimulates the renin–angiotensin–aldosterone system (RAAS). Adipocyte-released RAAS hormones and substances exacerbate RAAS, leading to high blood pressure via vasoconstriction and salt/water retention [72,73]. A study on a cohort of 100.000 children showed undoubtedly strong correlations between higher BMI and higher BP values, with the largest increase in BP levels being observed in the children who became obese earliest in life [74]. Moreover, the study showed that, compared to lean age-matched children, obese subjects were two times more likely to develop high BP, while severely obese children were over four times more likely to become hypertensive [71]. Obesity-related hypertension in children remains reversible for a long time [75,76], but its effects are cumulative with all the processes involved in atherosclerosis and metabolic impairment [77]. Our results are in line with previous findings; we showed strong positive correlations between clinical measurements such as BMI, WC, WHR, general fat mass, truncal fat mass, and both peripheral and central BP (Table 5 and Table 8). Measurements of central BP in children are cautiously regarded due to several studies which have suggested that the values obtained by tonometry and oscillometry devices may be underestimated [78,79,80]. Especially in children with risk factors for high BP, obtaining correct measurements is very important. A recent study on 1324 children aged from 6 to 8 years, using a Mobil-O-Graph device, showed that both weight and body fat mass are independently correlated with central BP and cPP levels, and recommended these measurements as valuable screening tools for CV risk assessment [81]. In a study involving 348 subjects aged from 8 to 18 years, severe obesity was associated with significantly higher central BP levels, compared to less critical weight excess [78]. 

As mentioned earlier, an important aspect of screening for obese patients at higher risk of developing metabolic and cardiovascular disorders is the assessment of abdominal adiposity. Measuring the waist circumference and calculating the waist-to-height ratio are crucial parts of the physical examination of obese children and should not be overlooked. Waist circumference, an acknowledged marker of insulin resistance [82], presented, in our study, a strong correlation with the truncal fat mass measured by body composition analysis through electrical impedance (ρ = 0.78, *p* < 0.0001). It is not only strongly correlated with HOMA-IR (ρ = 0.69, *p* < 0.0001), but also with every single vascular biomarker assessing atherosclerosis and arterial stiffness that has been included in the study (Table 5). WHR is considered a more sensible central adiposity marker, one less dependent on age than WC, with a cut-off value of 0.5 being validated for children ≥ 6 years old and adults [83,84], and a cut-off of 0.6 placing the child at risk for metabolic syndrome [48]. It is an anthropometric marker very reliably associated with visceral adiposity, being a better predictor of body fat percentage than are BMI and WC [85]. A cross-sectional study including 14.493 children aged from 5 to 18 years old showed that a rise in WHR was substantially linked with an increase in cardio-metabolic risk in overweight and obese participants: of obese children with a WHR ≥ 0.6, 32% had metabolic syndrome, 26% had raised non-HDL-c levels, 18% had elevated C-reactive protein levels, and 69% had elevated HOMA-IR values [86]. Our results showed strong correlations between WHR and HOMA-IR (ρ = 0.51, *p* < 0.0001), as well as with all the vascular biomarkers, except for DBP (Table 5). Both WC and WHR were shown to be independent predictors of peripheral and central BP levels (Table 7).

In normal-weight children and adolescents, CIMT is not influenced by sex or age [87], but in adults, men display higher CIMT than women [88]. PWV and central BP are more elevated in healthy prepubescent girls than in prepubescent boys, but once puberty is completed, boys present higher markers than girls [89]. These differences in PWV between the sexes in healthy children and young individuals are attributed to the protective roles of estrogen and progesterone [90]. Moreover, it seems that the fluctuation of hormones during the phases of the menstrual cycle has a particular role regarding arterial stiffness [91,92], and that the lower BP levels during the luteal phase could be one of the causes [90]. Although testosterone is a predictor of arterial stiffness in both males [90] and females [93], recent studies have recognized its positive effect on arterial elasticity [94] and reactivity [95] in men. These differences in arterial stiffness are seen in healthy, lean individuals. The pro-inflammatory activity of excess adipose tissue seems to partially erase the physiological influence of sex hormones, as obese children present both hormonal alterations and metabolic impairments [67]. Our study detected significantly higher CIMT in boys (*p* = 0.01) when assessing all boys versus all girls, but no difference when comparing only obese boys versus obese girls. No differences among the rest of the vascular biomarkers were attributed to sex. 

The lifelong atherosclerotic processes commence in early childhood,, as influenced by genetics and environment [96]. Age-related arterial stiffness is caused by collagen changes and reduced endothelial elastin [97]. Excess adiposity accelerates these processes. In children, age does not seem to be such a relevant factor in either the values of CIMT or PWV, as the inflammatory atherosclerotic processes caused by excessive weight affect the endothelial wall far more than age [9,40,98]. Our results showed significantly higher values for CIMT in children aged from 12 to 15 years than in those <12 years old (*p* = 0.02) and higher PWV and cSBP levels in > 15-year-olds compared to <12-year-olds (*p* = 0.01, and *p* = 0.02, respectively). No differences were detected across groups for the rest of the vascular biomarkers (Table 2). We can make a case, therefore, for affirming that older obese children present higher CIMT, PWV, and cSBP levels than do younger ones. However, in such young, obese individuals, age has a smaller impact on the arterial walls, which, in any case, is very difficult to estimate and separate from the processes caused by puberty in the presence of obesity [99,100,101]. 

Puberty itself does not cause atherosclerosis or arterial stiffness [67]. During puberty, the body is significantly less insulin sensitive due to hormonal and metabolic changes [102]. However, there is sufficient proof that children who start puberty being obese remain insulin-resistant post-puberty as well [102]. Our study has detected significantly higher HOMA-IR in obese pre- and post-pubertal subjects compared to their lean peers having the same Tanner stage, but not a significant difference between pubertal subjects of the two groups (Table 12). This goes to show that insulin resistance affects pubertal lean subjects as well. Contrary to our previous work, this study has shown significant differences between pre-pubertal, pubertal, and post-pubertal obese children with regard to the studied vascular biomarkers. We detected significantly higher values in post-pubertal children compared to pre-pubertal ones. Mostly, mean values for pre-pubertal children were significantly lower than in those who had started or finished puberty (Table 3). However, Tanner stages were not observed as independent predictors of CIMT, PWV, and AIx, but only of central BP values (Table 7). Excess androgens in obese girls could cause an accelerated atherosclerotic process, one resulting in higher CIMT and arterial stiffness [93], and this could also account for the differences between pre- and post-pubertal children.

In this study, we managed to focus our attention on the effect of insulin resistance on vascular biomarkers. The insulin resistance associated with obesity represents the greatest risk factor for developing type 2 diabetes and cardiovascular disorders in adulthood [103]. Insulin resistance reduces insulin’s ability to regulate glucose in muscles, adipose cells, and the liver [104]. It affects protein and lipid metabolism, endothelial function, and gene expression [104]. Increased acetyl-coenzyme-A production in the liver leads to adipose tissue inflammation [103]. Excessive fatty acids contribute to insulin resistance in obese children [103]. While euglycemic clamp and minimal-model tests are gold standards, they are invasive and used mainly in research [105]. The oral glucose tolerance test (OGTT) is reliable but time-consuming and less reproducible [106,107]. HOMA-IR is an easy and reliable biomarker for obese children, despite being more expensive than fasting glucose or hemoglobin A1c [105]. Insulin resistance varies based on puberty, race, sex, genetics, and environment [104], requiring adjusted HOMA-IR cut-offs. In our study, we used the cut-off values of HOMA-IR proposed in studies made with Caucasian subjects of similar ages to our subjects [61,62]. Our results not only point out the important association between HOMA-IR and weight excess (significant correlations with BMI, WC, and WHR) but also show extremely significant correlations to all vascular biomarkers (Table 11, Figure A7 and Figure A8). We also analyzed the correlation between HOMA-IR and the vascular biomarkers in subjects divided into puberty development stages; we detected significant correlations in the case of pre- and post-pubertal children, but not in the pubertal ones, most likely due to the relative insulin-resistant status of lean pubertal subjects (Table 13). Furthermore, HOMA-IR is an independent predictor for PWV, AIx, SBP, DBP, cSBP, and cDBP, as shown by the regression analysis depicted in Table 20.

We also looked at one of the clinical signs recognized to be a reliable sign of insulin resistance, acanthosis nigricans. HOMA-IR correlates reliably, but not perfectly, with the presence of acanthosis nigricans. The more severe the obesity and the higher the HOMA-IR, the more likely the presence of acanthosis nigricans [108,109,110,111]. In our study, 48% of the obese subjects displayed acanthosis nigricans. We evaluated the association between HOMA-IR and acanthosis nigricans according to Tanner stages, in order to improve the accuracy of our analysis. For pre-pubertal obese subjects, using a HOMA-IR cut-off value of 2.3 [61], out of the five children with a HOMA-IR ≥ 2.3, three presented acanthosis nigricans, while for those with a HOMA-IR < 2.3, out of seven subjects, only one presented acanthosis nigricans. Meanwhile, for pubertal and post-pubertal subjects, almost 40% presented a HOMA-IR above the set cut-off value of 3.4 [62]. Thirteen out of fifteen subjects with HOMA-IR ≥ 3.4 presented acanthosis nigricans. (See Table 14.) Further on, we looked at the possibility of predicting the presence of acanthosis nigricans by evaluating the vascular biomarkers. The AUC-ROC analysis showed optimal cut-off values for PWV at >4.8 m/s, SBP value > 125 mmHg, and a cSBP > 117 mmHg as predictive of the presence of acanthosis nigricans. (See Table 15.) CIMT has been shown to present significantly larger values in individuals with acanthosis nigricans [112]. Severe-grade acanthosis nigricans associates a larger PWV, but significance drops when adjusting for age, ethnic group, and BMI, which is the result of a study that suggests caution when interpreting the relevance of acanthosis nigricans for cardiovascular risk [113]. Our opinion is that obese children with acanthosis nigricans should be screened for metabolic and vascular alterations.

Given the intricate associations between insulin resistance, obesity-related hormonal alterations, and puberty itself, we analyzed the post-pubertal girls with regard to the possible menstrual cycle irregularities (a sign of PCOS) that can possibly be associated with increased atherosclerosis and arterial stiffness. It is not clear to what degree each of these pathological mechanisms contributes to vascular damage. PCOS is characterized by non-ovulatory menstrual cycles, hyperandrogenism, and insulin resistance. A recent study showed that individuals with PCOS displayed significantly larger CIMT compared to healthy controls of the same BMI and the same frequency of smoking. Moreover, a PCOS diagnosis was considered the strongest predictor of CIMT, even after adjusting for BMI, age, and smoking [114]. A study on obese adolescents with PCOS showed higher CIMT and lower arterial compliance in the PCOS group, as compared to obese girls [115]. Obese adolescents with PCOS who are also normotensive display higher carotid stiffness, and even left ventricular remodeling, which are both attributed to insulin resistance [116]. In contrast, another study suggests that the greater arterial stiffness in obese girls with PCOS is actually attributed to their central obesity, rather than to PCOS [117]. In our study, we identified twelve obese adolescents, six of whom had irregular menses. Our results showed that obese girls with irregular menses displayed significantly higher PWV, SBP, and DPB levels, but not higher CIMT or central BP levels (Table 15). These girls also presented higher HOMA-IR (mean value of 4.8), BMI, WC, and WHR, as well as a higher prevalence of acanthosis nigricans (Table 16). Further evaluations are, of course, needed, as a proper analysis needs far more subjects than ours had.

The results of the blood tests analysis included in this study strengthen our previous findings and complement them with new data. Of the significant differences in blood parameters between obese and controls, we have mentioned the higher LDL-c, TG, GPT, GOT, creatinine, uric acid, and lower ionized calcium detected in obese children (Table 18). Uric acid, a catabolic metabolite, is elevated in children with obesity, as a consequence of increased dietary intake of purines and due to the impaired glycolytic pathway, which results in accumulation of substrate for uric acid and the alterations of uric acid renal excretion, both caused by insulin resistance [118]. Uric acid in children with obesity is related to body composition, and its serum levels are lowered by weight loss [119]. Recent studies suggest that uric acid may serve as a target in the therapy of type 2 diabetes, due to the fact that elevated uric acid levels may be interpreted as a marker of risk for developing type 2 diabetes [120,121]. Our results show that serum uric acid is not only strongly correlated with PWV and peripheral and central BP levels (Table 19), but is an independent predictor of CIMT, PWV, SBP, and cPP (Table 20). These results are in line with other recent publications which have shown that the relation between uric acid and vascular stiffness in children is mediated by insulin resistance and BP levels [122], and that there is an association between high levels of uric acid and carotid atherosclerosis [123]. 

Having discussed the rest of the other serum parameters in our previous work [9,10], we will only reiterate the significant results, which strengthen our previous claims. LDL-c, probably the biggest contributor to atherosclerotic plaque [124], correlates with CIMT, and with arterial stiffness markers as well. HDL-c, an anti-inflammatory, protective lipoprotein [125] displaying low values in sedentary obese children [124], presented significant negative correlations with central BP levels in the present study. Non-HDL-c, a marker of atherosclerosis as reliable as LDL-c is [126], correlated significantly with PWV. High TG levels, a marker of metabolic syndrome caused by a chaotic and unhealthy lifestyle and diet and an important parameter for evaluating one’s cardiovascular risk [127], showed significant correlations, not only with PWV but with both peripheral and central systolic BP. The lipid ratios, LDL-c/HDL-c ratio, TG/HDL-c ratio, and TC/HDL-c ratio are reliable indicators of metabolic distress, as well as of dysglicemic and cardiovascular risk [128,129,130,131]. Although, in this study, we did not observe significant mean differences between the lipid ratios of obese vs. normal-weight children, in our previous work, we showed significant associations between the TG/HDL-c ratio and TC/HDL-c ratio, respectively, as well as the markers of arterial stiffness [10]. The only significant correlations the present study has detected with regard to lipid ratios was for the LDL-c/HDL-c ratio, which positively correlated with DBP and cDBP values (Table 19). The LDL-c/HDL-c ratio represents a predictor of cardiovascular risk, and can even be used in predicting glycemic control in patients with type 2 diabetes mellitus [131]. Efficiently managing the dyslipidemic lipid values in adolescence helps decrease the risk of high CIMT in young adulthood, because dyslipidemia secondary to obesity at adolescent age increases the risk of a higher CIMT as young adults, compared to adolescents who do not present both risk factors [132]. 

Elevated transaminase levels are very significant to vascular health [10,133]. This study showed that GPT correlates to CIMT, PWV, and central and peripheral systolic BP levels. GOT was correlated only with peripheral BP levels. 

25-OH vitamin D has been shown to interact with vascular vitamin D receptors of the endothelium, decreasing the proliferation of smooth cell muscles, and thus providing a cardiovascular protective role [134]. In obese children, vitamin D levels are usually low [135], and correcting their blood levels was proven to improve insulin sensitivity and lower BP levels [136]. Both 25-OH vitamin D and ionized calcium serum levels negatively correlated with PWV and peripheral and central BP levels in this study (Table 19). The lower ionized calcium levels are most likely associated with low vitamin D levels and the children’s unbalanced diet. 

The multilinear regression model detected GPT as a significant predictor of PWV, SBP, and cSBP, TC/HDL-c as a predictor of cDBP, and TG as a predictor of cPP. These results show that there is an intricate connection between the serum tests that are taken by clinicians on a daily basis and the vascular biomarkers which announce the deterioration of vascular health; hence, these correlations should determine clinicians’ practices to more properly detect patients at risk and to further investigate them more efficiently.

The key strength of this study is that the findings confirm the initial premise that obese children present detectable vascular disruption, although being almost completely asymptomatic. The highlight of our work is that we managed to integrate CIMT, a marker of subclinical atherosclerosis, and PWV, a marker of arterial stiffness, as well as peripheral and central BP levels, and not only analyze them all in multiple contexts but also propose cut-off values. To our knowledge, this makes our research unique, as other scientific papers on the subject have focused either on CIMT, or on arterial stiffness surrogate markers in the case of obese children. We were also successful in showing the importance of evaluating the insulin resistance status, by both clinical and laboratory methods, in painting a more accurate picture of an obese child’s cardiovascular risk. We showed that the insulin resistance status represents a crucial factor in the progression of vascular damage. We consider that failing to perform the flow-mediated dilation and liver elastography measurements are the greatest limitations of this study, but we plan on integrating these techniques in our future research. The number of subjects involved in the study could have been larger, but, according to our pre-statistical estimations, the ratios were sufficient to provide statistical significance. 

## 5. Conclusions

Excess weight in children represents a promoter of arterial stiffness and subclinical atherosclerosis starting at young ages, progressively aggravating the vascular disruptions and increasing the risk of developing cardiovascular disorders earlier in their adult life.

Waist circumference and waist-to-height ratio are crucial clinical markers, strongly correlated with CIMT, PWV, AIx, peripheral and central BP, and HOMA-IR.

Insulin-resistant obese children present increased arterial stiffness and subclinical atherosclerosis. HOMA-IR is an independent predictor of all the vascular biomarkers analyzed, except for CIMT. Acanthosis nigricans is a significant marker of vascular damage associated with insulin resistance; hence, the vascular health of the children displaying it should be more extensively investigated.

Increased levels of uric acid, LDL-c, non-LDL-c, TG, GOP, and GPT, and low levels of HDL-c and 25-OH vitamin D should enable clinicians to more accurately identify patients at risk and to further explore their vascular health.

We conclude that CIMT and the markers of arterial stiffness are non-invasive vascular biomarkers that could significantly elevate any clinician’s work with obese children. Providing a better understanding of how these patients develop irreversible cardiovascular damage, and at a time when these alterations are completely preventable, is an endeavor worth undertaking. Therefore, future prospective research is planned for these patients in order to detect how weight loss or progressive weight gain affect these vascular biomarkers.

## Figures and Tables

**Table 1:** Distribution of subjects by sex and age.

		Obese (*n* = 50)	%	Controls (*n* = 34)	%
Sex	Female	23	46	18	53
	Male	27	54	16	47
Age	<12 y	22	44	13	38
	12–15 y	17	34	13	38
	>15 y	11	22	8	24

**Table 2:** One-way ANOVA comparison of obese subjects according to age groups. The *p*-values in the comparative T-Student tests between groups were Bonferroni-adjusted in order to keep the significance threshold <0.05. The significant results in this table are in bold text.

		Mean Values			*p*-Values		
	ANOVA *p*-value	<12 y	12–15 y	>15 y	<12 y vs. 12–15 y	<12 y vs. >15 y	12–15 y vs. >15 y
CIMT	**0.02**	0.44	0.47	0.45	**0.02**	0.68	0.66
PWV	**0.01**	4.8	5	5.2	0.24	**0.01**	0.65
cSBP	**0.04**	115.4	118.94	125.45	0.84	**0.02**	0.5

**Table 3:** One-way ANOVA comparison of obese subjects according to Tanner-stage groups. The *p*-values in the comparative T-Student tests between groups were Bonferroni-adjusted in order to keep the significance threshold <0.05. The significant results in this table are in bold text.

		Mean Values			*p*-Values		
	ANOVA *p*-value	Tanner 1	Tanner 2–4	Tanner 5	Tanner 1 vs. Tanner 2–4	Tanner 1 vs. Tanner 5	Tanner 2–4 vs. Tanner 5
CIMT	**0.0009**	0.42	0.44	0.46	0.39	**0.002**	**0.04**
PWV	**0.002**	4.5	4.6	5	0.9	**0.003**	**0.001**
AIx	**0.008**	23.31	26.39	31.58	0.25	**0.004**	**0.02**
SBP	**0.003**	117.81	119.6	127.82	0.95	**0.02**	**0.003**
DBP	**0.01**	77.86	75	81.9	0.75	**0.36**	**0.003**
MAP	**0.002**	97.84	97.3	104.86	0.96	0.057	**0.0006**
cSBP	**0.004**	106.22	111.33	118.24	0.42	**0.01**	0.054
cDBP	**0.09**	73.4	71.78	77.03	0.97	0.69	**0.04**
cPP	**0.005**	32.81	39.54	41.2	**0.02**	**0.003**	0.98
HR	0.21	88.6	84.2	83	0.12	0.09	0.45

**Table 4:** Differences between the two study groups for BMI, WC, and WHR.

	BMI (kg/m^2^)		WC (cm)		WHR	
	Mean	SD	Mean	SD	Mean	SD
Obese	29.23	6.78	99.41	16.68	0.62	0.09
Controls	19.01	2.48	65.41	8.96	0.43	0.03
*p*-value	<0.0001		<0.0001		<0.0001	

**Table 5:** The vascular biomarkers in correlation to the BMI, WC, and WHR (Spearman’s correlations) in all patients (*n* = 84).

	Correlations	CIMT	PWV	AIx	SPB	DBP	MAP	cSBP	cDBP	cPP	HR
BMI	ρ	0.52	0.7	0.36	0.7	0.32	0.56	0.75	0.52	0.49	0.37
	*p*-value	<0.0001	<0.0001	0.0007	<0.0001	0.003	<0.0001	<0.0001	<0.0001	<0.0001	0.04
WC	ρ	0.53	0.73	0.39	0.72	0.29	0.55	0.76	0.51	0.5	0.24
	*p*-value	<0.0001	<0.0001	0.0002	<0.0001	0.007	<0.0001	<0.0001	<0.0001	<0.0001	0.21
WHR	ρ	0.38	0.58	0.33	0.58	0.2	0.42	0.64	0.38	0.43	0.13
	*p*-value	0.0004	<0.0001	0.001	<0.0001	0.06	0.0001	<0.0001	0.0004	<0.0001	0.27

**Table 6:** The multiple regression models with clinical parameters as independent variables (W, BMI, WC, WHR, Tanner stages, and acanthosis nigricans).

Dependent Variable	*F*	*p*-Value	*R^2^*	Variance %
CIMT	6.39	<0.001	0.44	43.74
PWV	14.68	<0.001	0.64	64.09
AIx	2.7	0.008	0.25	24.74
SBP	13.89	<0.001	0.63	62.81
DBP	3.28	0.002	0.29	28.55
MAP	9.43	<0.001	0.53	53.42
cSBP	16.86	<0.001	0.67	67.21
cDBP	6.46	<0.001	0.44	43.98
cPP	3.63	<0.001	0.31	30.62
HR	0.61	0.78	0.07	6.92

**Table 7:** Multiple regression analysis for clinical parameters (Tanner, W, BMI, WC, WHR, and acanthosis nigricans) as predictors of vascular biomarkers.

Dependent Variable	Independent Variable	B Coefficient	Beta Coefficient	Std. Error	t	*p*-Value
SBP	WC	1.52	2.77	0.6	2.51	0.01
	WHR	−204.26	−2.11	89.23	−2.29	0.02
DBP	BMI	2.17	1.88	0.99	2.19	0.03
	WC	1.32	3.17	0.64	2.08	0.04
	WHR	−216.47	−2.94	93.89	−2.31	0.02
MAP	BMI	1.9	1.58	0.83	2.28	0.02
	WC	1.42	3.27	0.53	2.65	0.01
	WHR	−210.36	−2.75	78.95	−2.66	0.009
cSBP	WC	1.7	2.8	0.63	2.71	0.008
	WHR	−225.68	−2.11	92.71	−2.43	0.01
	Tanner	7.13	0.24	3.52	2.02	0.04
cDBP	WHR	−179.4	−2.31	87.96	−2.04	0.04
	Tanner	9.88	0.45	3.34	2.95	0.004

**Table 8:** Correlations between the vascular biomarkers and body composition parameters in obese patients (Pearson’s r or Spearman’s ρ, according to the normality of the data).

Vascular Biomarkers	Correlations	Fat Mass (kg)	Trunk Fat Mass (kg)	Muscle Mass (kg)	Body Water (kg)
CIMT	ρ/r	0.39	0.41	0.41	0.4
	*p*-value	0.005	0.002	0.003	0.004
PWV	ρ/r	0.65	0.6	0.48	0.47
	*p*-value	<0.0001	<0.0001	0.0004	0.0005
AIx	ρ/r	0.34	0.32	0.17	0.19
	*p*-value	0.01	0.02	0.22	0.18
SBP	ρ/r	0.62	0.53	0.41	0.4
	*p*-value	<0.0001	0.0001	0.003	0.004
DBP	ρ/r	0.42	0.29	0.24	0.24
	*p*-value	0.002	0.04	0.08	0.08
MAP	ρ/r	0.55	0.44	0.38	0.37
	*p*-value	<0.0001	0.001	0.006	0.007
cSBP	ρ/r	0.63	0.55	0.46	0.45
	*p*-value	<0.0001	<0.0001	0.0006	0.0009
cDBP	ρ/r	0.48	0.38	0.33	0.33
	*p*-value	0.0004	0.006	0.01	0.01
cPP	ρ/r	0.18	0.23	0.18	0.18
	*p*-value	0.19	0.1	0.19	0.2
HR	ρ/r	0.38	0.47	0.26	0.24
	*p*-value	0.003	0.02	0.12	0.18

**Table 9:** AUC-ROC analysis for each vascular biomarker with the presence of obesity considered the pathological instance.

	Criterion (Cut-Off)	AUC	*p*	Se %	95% CI	Sp %	95% CI	PPV %	NPV %
CIMT	>0.4	0.69	0.002	98	89.4–99.9	41.2	24.6–59.3	71	93.3
PWV	>4.6	0.78	<0.0001	72	57.5–83.8	73.5	55.6–87.1	79.61	64.1
AIx	>31	0.64	0.02	46	31.8–60.7	76.5	58.8–89.3	74.22	49.13
SBP	>119	0.79	<0.0001	64	49.2–77.1	82.3	65.5–93.2	84.13	60.81
DBP	>84	0.53	0.62	36	22.9–50.8	91.2	76.3–98.1	85.73	49.23
MAP	>102.5	0.67	0.003	44	30–58.7	85.3	68.9–95	91.47	50.9
cSBP	>106	0.86	<0.0001	90	78.2–96.7	67.6	49.5–82.6	80.31	82.14
cDBP	>76	0.71	0.0001	50	35.5–64.5	82.3	65.5–93.2	80.58	52.83
cPP	>36	0.77	<0.0001	76	61.8–86.9	70.6	52.5–84.9	79.15	66.68

**Table 10:** AUC-ROC analysis for each vascular biomarker, according to age groups, with the presence of obesity considered the pathological instance. The table depicts only the significant results of the analysis.

Age Group	Vascular Biomarker	Criterion (Cut-Off)	AUC	*p*	Se %	Sp %	PPV %	NPV %
<12 y	CIMT	>0.4	0.83	0.0001	100	61.5	81.42	100
	PWV	>4.6	0.8	0.001	72.75	84.6	88.85	64.73
	SBP	>114	0.86	<0.0001	90.9	69.2	83.28	81.83
	MAP	>93.5	0.74	0.01	90.9	61.5	81.21	80.76
	cSBP	>106	0.91	<0.0001	95.45	84.6	91.27	91.67
	cPP	>34	0.88	<0.0001	81.8	94.6	89.96	73.35
12–15 y	CIMT	>0.46	0.84	<0.0001	52.9	100	100	61.89
	PWV	>4.5	0.86	<0.0001	94.1	69.2	80	90
	AIx	>31	0.68	0.06	52.9	92.3	90	60
	SBP	>124	0.71	0.03	52.9	100	100	61.9
	cSBP	>115	0.86	<0.0001	64.7	100	100	68.4
	cDBP	>75	0.77	0.001	64.7	84.6	84.6	64.7
	cPP	>36	0.71	0.03	76.5	69.2	76.5	69.2
>15 y	PWV	>5	0.74	0.04	81.8	62.5	75	71.4
	SBP	>126	0.82	0.0008	81.8	75	81.8	75
	cSBP	>123	0.88	<0.0001	72.7	100	100	72.7
	cDBP	>79	0.76	0.02	63.6	87.5	87.5	63.6
	cPP	>44	0.76	0.03	63.6	100	100	66.7

**Table 11:** Correlations between the HOMA-IR and vascular markers (Spearman’s correlation) in all patients (*n* = 84).

	CIMT	PWV	AIx	SPB	DBP	MAP	cSBP	cDBP	cPP	HR
ρ	0.44	0.64	0.4	0.63	0.43	0.6	0.59	0.47	0.34	0.22
*p*-value	<0.0001	<0.0001	0.0001	<0.0001	<0.0001	<0.0001	<0.0001	<0.0001	0.001	0.17

**Table 12:** Descriptive and comparative data regarding the HOMA-IR, according to Tanner stages.

Tanner Stage	Group	*n*	Boys	Girls	Mean Age	SD	Age Limits	HOMA-IR Median Values	SD	*p*-Value
1	Obese	12	9	3	8.6	1.6	6–11 y	1.85	3.9	0.01
	Controls	10	6	4	7.6	1.42	6–10 y	1	0.25	
2 3 4	Obese	20	12	8	11.6	1.6	9–15.5 y	1.8	1.49	0.34
	Controls	13	6	7	12	1.52	9–14 y	1.63	0.2	
5	Obese	18	6	12	16	1.26	14–18 y	3.91	1.39	0.002
	Controls	11	4	7	16	1.26	14–18 y	2.2	0.35	

**Table 13:** Correlations between HOMA-IR and the vascular markers, according to Tanner stages (Spearman’s correlations).

		CIMT	PWV	AIx	SPB	DBP	MAP	cSBP	cDBP	cPP	HR
Tanner 1 (*n* = 22)	ρ	0.39	0.61	0.56	0.67	0.45	0.64	0.66	0.43	0.52	0.16
	*p*-value	0.07	**0.002**	**0.006**	**0.0007**	**0.03**	**0.01**	**0.0007**	**0.04**	**0.01**	0.34
Tanner 2, 3, 4 (*n* = 33)	ρ	0.13	0.22	−0.08	0.15	0.02	0.17	0.15	0.16	0.11	0.13
	*p*-value	0.46	0.2	0.62	0.4	0.9	0.32	0.37	0.37	0.52	0.27
Tanner 5 (*n*=29)	ρ	0.31	0.66	0.43	0.68	0.57	0.7	0.68	0.66	0.23	0.09
	*p*-value	0.09	**0.0001**	**0.02**	**<0.0001**	**0.001**	**<0.0001**	**<0.0001**	**0.0001**	0.22	0.45

The significant results in this table are in bold text.

**Table 14:** Number of obese subject distribution according to HOMA-IR cut-off values and the presence of acanthosis nigricans.

HOMA-IR Cut-Off Values in Obese Subjects	Puberty Development	*n*	*n* of Patients with HOMA-IR >Cut-Off	Acanthosis Nigricans Present in:	*n* Of Patients With HOMA-IR <Cut-Off	Acanthosis Nigricans Present in:
2.3	Pre-pubertal	12	5	3	7	1
3.4	Pubertal and post-pubertal	38	15	13	23	7

**Table 15:** AUC-ROC analysis for each vascular biomarker, with the presence of acanthosis nigricans consideredthe pathological instance.

	Criterion (Cut-Off)	AUC	*p*	Se %	95% CI	Sp %	95% CI	PPV %	NPV %
CIMT	>0.47	0.67	0.02	50	29.1–70.9	88.4	69.8–97.6	86.36	54.61
PWV	>4.8	0.77	0.0001	75	53.3–90.2	80.8	60.6–93.4	85.14	68.74
AIx	>28	0.58	0.31	66.7	44.7–84.4	53.8	33.4–73.4	67.95	52.37
SBP	>125	0.79	<0.0001	75	53.3–90.2	84.6	65.1–95.6	87.73	69.72
DBP	>72	0.73	0.001	87.5	67.6–97.3	53.8	33.4–73.4	73.56	74.55
MAP	>97	0.82	<0.0001	91.7	73–99	65.4	44.3–82.8	79.56	84.28
cSBP	>117	0.8	>0.0001	70.8	48.9–87.4	80.8	60.6–93.4	84.41	65.32
cDBP	>74	0.7	0.007	79.2	57.8–92.9	65.4	44.3–82.8	77.07	68.15
cPP	>43	0.6	0.22	54.2	32.8–74.4	76.9	56.4–91	77.51	53.33

**Table 16:** Comparative data of the subgroups created according to the menstrual cycles for Tanner stage 5 girls.

	Menstrual Cycles		*n*	Mean HOMA-IR	Acanthosis Nigricans Present In:	BMI kg/m^2^	WC cm	WHR
Obese		Regular	6	3.43	3 girls	33.45	105	0.65
		Irregular	6	4.8	5 girls	40.56	121	0.76
Controls		Regular	7	2.01	No girl	20.29	70.14	0.42

**Table 17:** Comparisons between the values of vascular biomarkers of Tanner stage 5 obese girls with regular and irregular menses.

	CIMT	PWV	AIx	SPB	DBP	MAP	cSBP	cDBP	cPP
Regular menses	0.43	4.76	28.16	122.83	77	99.91	114.33	77.33	37
Irregular menses	0.48	5.38	37.16	135.33	89.33	112.33	126.83	82.66	44.16
*p*-value	0.06	**0.002**	0.09	**0.006**	**0.01**	**0.003**	**0.009**	0.27	0.26

The significant results in this table are in bold text.

**Table 18:** Comparisons between the blood biomarkers in the obese vs. controls.

Variables	Obese Subjects		Normal-Weight Subjects		
	Mean/Median	SD	Mean/Median	SD	*p*-Value
Fasting glucose	81	12.71	80.6	10.1	0.6
HDL-c	42.7	7.95	41	10.25	0.8
LDL-c	109.8	33.82	85.55	24.86	**0.04**
TC	174.28	33.83	160.76	27.62	0.06
TG	116.27	45.15	81	47.67	**0.02**
Non-HDL-c	131.56	36.62	118.26	27.92	0.07
LDL-c/HDL-c	2.37	1.27	2.5	0.77	0.67
TG/HDL-c	2.91	1.47	2	1.31	0.1
TC/HDL-c	3.9	1.4	3.7	0.88	0.42
GPT	30.5	15.07	24	10.85	**0.003**
GOT	27	10.98	21	8.74	**0.005**
Creatinine	0.52	0.13	0.4	0.1	**0.01**
Uric acid	4.9	1.44	3.88	0.74	**0.0001**
TSH	3.5	1.87	3.47	0.88	0.77
FreeT4	14.99	1.69	15.06	1.67	0.86
8 am cortisol	17.22	5.09	17	2.14	0.88
Ionized calcium	3.93	0.43	4.1	0.2	**0.009**
25-OH-vitamin D	19.84	9.93	24	8.4	0.22

The significant results in this table are in bold text.

**Table 19:** Correlations between the blood parameters and the vascular biomarkers in obese children.

Correlations	CIMT	PWV	AIx	SBP	DBP	MAP	cSBP	cDBP	cPP	HR
Fasting glucose	−0.19	−0.11	0.05	0.04	0.21	0.16	−0.001	0.27	−0.25	0.03
*p*-value	0.17	0.53	0.69	0.77	0.13	0.26	0.99	0.06	0.07	0.89
Uric acid	0.27	**0.41**	0.09	**0.35**	**0.32**	**0.4**	**0.46**	**0.33**	0.12	0.16
*p*-value	0.05	**0.02**	0.53	**0.01**	**0.02**	**0.04**	**0.0008**	**0.01**	0.4	0.78
Creatinine	0.14	0.24	0.09	0.14	0.01	0.08	0.16	0.02	0.15	0.04
*p*-value	0.33	0.08	0.49	0.31	0.93	0.55	0.24	0.85	0.29	0.91
HDL-c	−0.02	−0.2	0.07	−0.21	−0.19	−0.21	**−** **0.36**	**−** **0.34**	0.1	0.16
*p*-value	0.86	0.15	0.62	0.14	0.16	0.13	**0.02**	**0.01**	0.42	0.64
LDL-c	**0.31**	**0.39**	0.06	**0.42**	0.21	0.24	**0.36**	0.2	0.19	0.07
*p*-value	**0.03**	**0.02**	0.65	**0.02**	0.09	0.07	**0.03**	0.16	0.16	0.94
Total cholesterol	0.21	0.24	0.1	0.22	0.06	0.15	0.17	0.03	0.25	0.08
*p*-value	0.27	0.15	0.52	0.08	0.9	0.27	0.24	0.87	0.14	0.69
TG	0.27	**0.47**	0.16	**0.32**	0.15	0.22	**0.38**	0.09	0.06	0.04
*p*-value	0.07	**0.01**	0.34	**0.04**	0.22	0.17	**0.02**	0.66	0.79	0.78
Non-HDL-c	0.24	**0.33**	0.15	0.22	0.14	0.16	0.17	0.18	0.21	0.004
*p*-value	0.09	**0.03**	0.32	0.12	0.33	0.35	0.26	0.22	0.13	0.96
LDL-c/HDL-c ratio	0.14	0.17	0.05	0.12	**0.22**	0.16	0.09	**0.23**	0.08	0.18
*p*-value	0.2	0.12	0.64	0.27	**0.04**	0.13	0.4	**0.03**	0.43	0.09
TG/HDL-c ratio	0.19	0.23	0.11	0.18	0.09	0.12	0.15	0.19	0.08	0.01
*p*-value	0.22	0.1	0.43	0.34	0.53	0.46	0.39	0.17	0.54	0.57
TC/HDL-c ratio	0.1	0.04	0.1	0.04	0.17	0.1	0.05	0.24	0.23	−0.07
*p*-value	0.9	0.76	0.49	0.78	0.21	0.45	0.73	0.09	0.11	0.66
GPT	**0.31**	**0.48**	0.16	**0.35**	**0.55**	**0.44**	0.19	0.2	0.21	0.21
*p*-value	**0.03**	**0.001**	0.19	**0.03**	**0.001**	**0.01**	0.12	0.16	0.16	0.29
GOT	0.09	0.25	0.02	**0.31**	**0.42**	**0.37**	0.17	0.16	**0.28**	−0.13
*p*-value	0.54	0.17	0.54	**0.004**	**0.003**	**0.02**	0.92	0.25	**0.04**	0.48
8 a.m. cortisol	**0.28**	0.16	0.23	0.11	0.21	0.19	0.07	0.2	−0.13	−0.06
*p*-value	**0.04**	0.25	0.1	0.41	0.12	0.17	0.61	0.15	0.36	0.72
TSH	0.12	−0.7	−0.5	−0.004	−0.2	−0.11	0.05	−0.15	**0.34**	−0.03
*p*-value	0.4	0.63	0.7	0.97	0.14	0.44	0.71	0.3	**0.04**	0.88
FreeT4	0.17	0.16	0.08	0.16	0.06	0.13	0.09	0.1	0.02	0.09
*p*-value	0.21	0.26	0.55	0.26	0.66	0.36	0.53	0.47	0.87	0.59
25-OH- vitamin D	−0.19	**−** **0.41**	−0.09	**−** **0.37**	**−** **0.44**	**−** **0.43**	**−** **0.35**	**−** **0.42**	0.1	0.12
*p*-value	0.17	**0.003**	0.51	**0.007**	**0.001**	**0.001**	**0.01**	**0.002**	0.47	0.65
Ionized calcium	−0.2	**−** **0.35**	0.01	**−** **0.38**	**−** **0.35**	**−** **0.39**	**−** **0.39**	**−** **0.32**	−0.03	0.11
*p*-value	0.14	**0.01**	0.9	**0.006**	**0.01**	**0.005**	**0.004**	**0.02**	0.8	0.28

The significant results in this table are in bold text.

**Table 20:** Multiple regression analysis for blood parameters and HOMA-IR as predictors of vascular biomarkers.

Dependent Variable	Independent Variable	B Coefficient	Beta Coefficient	Std. Error	t	*p*-Value
CIMT	uric acid	0.009	0.09	0.003	2.68	0.008
PWV	HOMA-IR	0.1	0.41	0.02	4.4	<0.0001
	GPT	0.008	0.28	0.003	2.74	0.007
	uric acid	0.07	0.19	0.03	2.21	0.02
AIx	HOMA-IR	1.37	0.27	0.52	2.95	0.004
SBP	HOMA-IR	3.36	0.51	0.53	6.3	<0.0001
	GPT	0.18	0.31	0.07	2.44	0.01
DBP	HOMA-IR	1.92	0.37	0.46	4.15	0.0001
MAP	HOMA-IR	2.83	0.5	0.42	6.63	<0.0001
cSBP	HOMA-IR	2.71	0.41	0.65	4.17	0.0001
	GPT	0.18	0.27	0.08	2.11	0.03
	uric acid	2.19	0.29	0.95	2.29	0.02
cDBP	HOMA-IR	2.15	0.37	0.46	4.63	<0.0001
	TC/HDL-c ratio	1.87	1.72	0.74	2.51	0.01
cPP	uric acid	1.91	0.24	0.78	2.44	0.01
	TG	0.45	2.16	0.18	2.53	0.01

## Data Availability

Only the corresponding author may provide access to the data reported in this study. Due to privacy limitations, the data are not publicly accessible.

## Abbreviations

AIx	Index of augmentation
AUC	Area under the receiver operating characteristic curve
BMI	Body mass index
BP	Blood pressure
cDBP	Central diastolic blood pressure
CIMT	Carotid intima-media thickness
cPP	Central pulse pressure
cSBP	Central systolic blood pressure
DBP	Diastolic blood pressure
GOT	Aspartate aminotransferase
GPT	Alanine aminotransferase
H	Height
HDL-c	High-density lipoprotein cholesterol
HOMA-IR	Homeostatic model assessment for insulin resistance
HR	Heart rate
LDL-c	Low-density lipoprotein cholesterol
MAP	Mean arterial pressure
*n*	Number of subjects
PCOS	Polycystic ovary syndrome
PWV	Pulse wave velocity
ROC	Receiver operating characteristic
SBP	Systolic blood pressure
SD	Standard deviation
Se	Sensitivity
Sp	Specificity
Std.	Standard
TC	Total cholesterol
TG	Triglycerides
vs.	Versus
W	Weight
WC	Waist circumference
WHR	Waist-to-height ratio
y	Years
